# Supplementary material for: Utility of polygenic scores across diverse diseases in a hospital cohort for predictive modeling
Source: Nat Commun. 2024 Apr 12;15:3168. doi: 10.1038/s41467-024-47472-5 (PMC11014845; doi:10.1038/s41467-024-47472-5)
Supplement: Supplementary file 1 — Supplementary Information [file 41467_2024_47472_MOESM1_ESM.pdf]

Utility of Polygenic Scores Across Diverse Diseases in a Hospital Cohort for Predictive Modeling

Ting-Hsuan Sun[a], Chia-Chun Wang[a], Ting-Yuan Liu[b], Shih-Chang Lo[a], Yi-Xuan Huang[a], Shang-Yu Chien[a], Yu-De Chu[a], Fuu-Jen Tsai [c-f,†,*], Kai-Cheng Hsu[a, g, h,†,*]

[a] Artificial Intelligence Center, China Medical University Hospital, Taichung, 40447, Taiwan.

[b] Million-person precision medicine initiative, Department of Medical Research, China Medical University Hospital, Taichung, 40447, Taiwan.

[c] Department of Medical Research, China Medical University Hospital, Taichung, 40447, Taiwan.

[d] School of Chinese Medicine, China Medical University, Taichung, 40402, Taiwan.

[e] Division of Pediatric Genetics, Children's Hospital of China Medical University, Taichung, 40447, Taiwan.

[f] Department of Biotechnology and Bioinformatics, Asia University, Taichung, 41354, Taiwan.

[g] Department of Neurology, China Medical University Hospital, Taichung, 40447, Taiwan.

[h] Department of Medicine, China Medical University, Taichung, 40402, Taiwan.

[†] These authors contributed equally to this work.

[*] Corresponding author: Kai-Cheng Hsu (e-mail: kaichenghsu66@gmail.com) and Fuu-Jen Tsai (e-mail: 000704@tool.caaumed.org.tw)

Supplementary Results

The effect of case control matching ratio on the statistical value of PGS distribution

Given the extremely small P-values in the Wilcoxon rank sum test, we chose to transform all P-values into their corresponding $-log_{10}(P)$ to enhance observability. Upon investigation of 1730 phenotype−PGS pairs, Pearson correlation analyses revealed that the $-log_{10}(P)$ between 4X matching, 6X matching, and 8X matching all exceeded 0.992 (Supplementary Figure 1A). Only the 2X matching category displayed a trend where a greater difference in pairing proportions led to decreased correlation: the Pearson correlation between 2X matching and 4X matching was 0.984, between 2X matching and 6X matching was 0.976, and between 2X matching and 8X matching was 0.968.

When the phenotype−PGS pairs were stratified into four groups based on $-log_{10}(P)$ intervals (Supplementary Figure 1B), the first group, comprising phenotype−PGS pairs with $-log_{10}(P)$ >30,

encompassed 216 phenotype−PGS pairs. In this group, the $-log_{10}(P)$ for 4X matching, 6X matching, and 8X matching remained highly similar, with Pearson correlation coefficients all exceeding 0.981. However, the similarity between 2X matching and the other groups dropped to below 0.965.

The second group, with $-log_{10}(P)$ ranging between 15-30 and totaling 188 phenotype−PGS pairs, exhibited a strong correlation between 6X matching and 8X matching, with a Pearson correlation of 0.986. Conversely, the $-log_{10}(P)$ similarity between 4X matching and 6X matching, as well as 4X matching and 8X matching, sharply declined to 0.799 and 0.787, respectively. Interestingly, these values were even lower than the $-log_{10}(P)$ similarity between 2X matching and the latter two groups.

The third group, which featured $-log_{10}(P)$ between 5.602 and 15, comprising 324 phenotype−PGS pairs ($-log_{10}(2 \times 10^{-6}) = 5.602$). In this group, a robust correlation persisted between 6X matching and 8X matching, with a Pearson correlation of 0.982. Additionally, the $-log_{10}(P)$ similarity between 4X matching and 6X matching, as well as 4X matching and 8X matching, showed a slight rebound, reaching 0.841 and 0.848, respectively.

The fourth group consisted of phenotype−PGS pairs with $-log_{10}(P)$ of 5.602 or less, totaling 1002 pairs. Within this group, a substantial correlation endured between 6X matching and 8X matching, with a Pearson correlation of 0.987. The $-log_{10}(P)$ similarity between 4X matching and 6X matching, as well as 4X matching and 8X matching, continued to rise, reaching 0.874 and 0.886, respectively.

Finally, for each phenotype, we selected the PGS trait and the matching method with the highest $-log_{10}(P)$ (lowest P-value) for statistical analysis (Supplementary Figure 1C). The 8X matching method exhibited superior results in the first three groups, with 110/216 pairs, 81/188 pairs, and 132/324 pairs. In contrast, only in the fourth group did the number of 4X matching method (525/1002 pairs) surpass that of 8X matching method (168/1002 pairs). Therefore, we choose 8X matching for subsequent model development and performance evaluation.

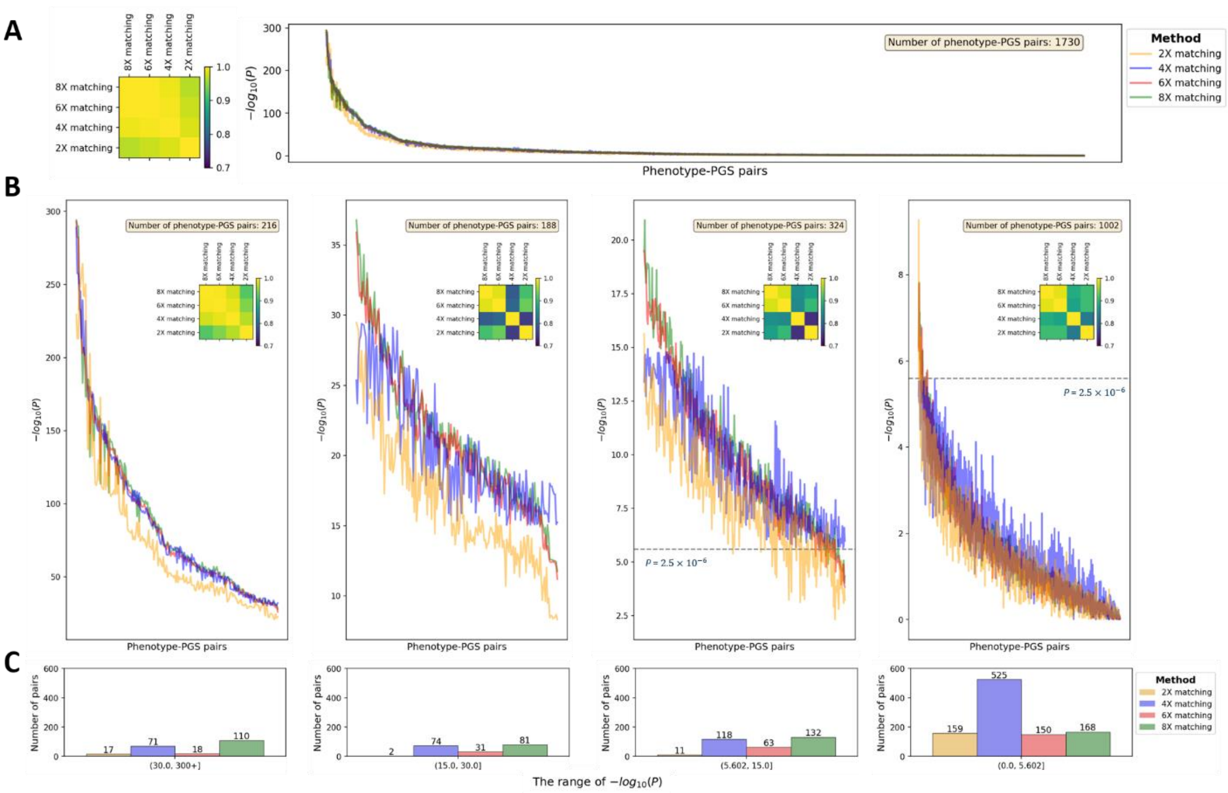

Supplementary Figure 1. Correlation analysis of Wilcoxon rank sum test results between different sample selection methods. (A) Calculated with 1730 phenotype and PGS trait phenotype−PGS pairs. (B) Strategic calculation by $-log_{10}(P)$ intervals. (C) Count results for the optimal pairing method based on $-log_{10}(P)$ for each phenotype.
