# Supplementary material for: Utility of polygenic scores across diverse diseases in a hospital cohort for predictive modeling
Source: Nat Commun. 2024 Apr 12;15:3168. doi: 10.1038/s41467-024-47472-5 (PMC11014845; doi:10.1038/s41467-024-47472-5)
Supplement: Supplementary file 3 — Description of Additional Supplementary Files [file 41467_2024_47472_MOESM3_ESM.pdf]

Description of Additional Supplementary Files

File Name: Supplementary Data 1
Description: The summary of case/control samples in phenotype traits.

File Name: Supplementary Data 2
Description: The pairing results of phenotype and PGS traits.

File Name: Supplementary Software 1
Description: Code used in PGS calculations and model construction.
