# Supplementary material for: Utility of polygenic scores across diverse diseases in a hospital cohort for predictive modeling
Source: Nat Commun. 2024 Apr 12;15:3168. doi: 10.1038/s41467-024-47472-5 (PMC11014845; doi:10.1038/s41467-024-47472-5)
Supplement: Supplementary file 7 — Reporting Summary [file 41467_2024_47472_MOESM7_ESM.pdf]

nature portfolio

Corresponding author(s): Fuu-Jen Tsai
Kai-Cheng Hsu

Last updated by author(s): Mar 7, 2024

Reporting Summary

Nature Portfolio wishes to improve the reproducibility of the work that we publish. This form provides structure for consistency and transparency in reporting. For further information on Nature Portfolio policies, see our Editorial Policies and the Editorial Policy Checklist.

Statistics

For all statistical analyses, confirm that the following items are present in the figure legend, table legend, main text, or Methods section.

n/a	Confirmed	
☐	☒	The exact sample size (*n*) for each experimental group/condition, given as a discrete number and unit of measurement
☐	☒	A statement on whether measurements were taken from distinct samples or whether the same sample was measured repeatedly
☐	☒	The statistical test(s) used AND whether they are one- or two-sided<br>*Only common tests should be described solely by name; describe more complex techniques in the Methods section.*
☐	☒	A description of all covariates tested
☐	☒	A description of any assumptions or corrections, such as tests of normality and adjustment for multiple comparisons
☐	☒	A full description of the statistical parameters including central tendency (e.g. means) or other basic estimates (e.g. regression coefficient) AND variation (e.g. standard deviation) or associated estimates of uncertainty (e.g. confidence intervals)
☐	☒	For null hypothesis testing, the test statistic (e.g. *F*, *t*, *r*) with confidence intervals, effect sizes, degrees of freedom and *P* value noted<br>*Give P values as exact values whenever suitable.*
☐	☒	For Bayesian analysis, information on the choice of priors and Markov chain Monte Carlo settings
☐	☒	For hierarchical and complex designs, identification of the appropriate level for tests and full reporting of outcomes
☐	☒	Estimates of effect sizes (e.g. Cohen's *d*, Pearson's *r*), indicating how they were calculated

Our web collection on statistics for biologists contains articles on many of the points above.

Software and code

Policy information about availability of computer code

Data collection	1. The PGS score files were download through FTP site by the wget command under Linux<br>2. The genotype calling process was conducted in accordance with guidelines and regulations provided by Affymetrix
Data analysis	1. The PGSs were calculated using PRSice-2 (https://choishingwan.github.io/PRSice/)<br>2. The phenotypes were identified through the createPhenotypes function in PheWAS (https://github.com/PheWAS/PheWAS)<br>3. The surjective pairing method was employed to link the phenotypes and PGS traits using the BeautifulSoup function (https://git.launchpad.net/beautifulsoup)<br>4. A two-sided Welch's two-sample t-test was calculated with the t.test() R function<br>5. A two-sided Wilcoxon rank sum test was calculated with the wilcox.test() R function<br>6. The logistic regression model with 6 evaluation metrics was performed with glm in R function

For manuscripts utilizing custom algorithms or software that are central to the research but not yet described in published literature, software must be made available to editors and reviewers. We strongly encourage code deposition in a community repository (e.g. GitHub). See the Nature Portfolio guidelines for submitting code & software for further information.

Data

Policy information about availability of data

All manuscripts must include a data availability statement. This statement should provide the following information, where applicable:

- Accession codes, unique identifiers, or web links for publicly available datasets
- A description of any restrictions on data availability
- For clinical datasets or third party data, please ensure that the statement adheres to our policy

The raw SNP array data are protected and are not available due to data privacy laws. However, we are committed to fostering collaboration and promoting transparency in scientific research. As such, we welcome collaborative projects and discussions with fellow researchers who may require access to the data. Please provide your identity, employer, purpose of data access, and IRB approval to the mailbox of the corresponding author, Dr. Fuu-Jen Tsai (000704@tool.caaumed.org.tw). We will meet within one month to discuss and respond. The statistical results generated in this study are provided on the GeneAnaBase website, which can be found at https://pgscatalog.azure.nihxcmuh.org/#/.
All codes used for data download, processing, calculation, and graphing are recorded in the Supplementary Software. Please refer to this document for detailed information.

Research involving human participants, their data, or biological material

Policy information about studies with human participants or human data. See also policy information about sex, gender (identity/presentation), and sexual orientation and race, ethnicity and racism.

Reporting on sex and gender

In this article, we adhere to a clear distinction between "sex" as a biological attribute and "gender" as a characteristic influenced by social and cultural circumstances. The definition of "sex" in this study is derived from the sex chromosome types detected by SNP array data, which serves as clinical data for statistical purposes.

All analytical methods employed in this research are conducted in strict accordance with the guidelines and regulations provided by Affymetrix, ensuring the robust and consistent handling of sex-related data.

When combining clinical data, the recorded gender on individual identification (ID) numbers is cross-referenced with the gender information recorded in the electronic health record (EHR) system. For data to be included in subsequent analyses, it is imperative that these three sources —biological sex, gender on ID numbers, and gender in the EHR system—are in complete agreement. Any discrepancies are investigated and resolved to maintain data integrity.

Reporting on race, ethnicity, or other socially relevant groupings

The SNP array data used in this article were sourced from the Million Person Precision Medicine Initiative in China Medical University Hospital. In this study, the socially relevant categorization variable employed pertains to ethnicity, specifically East Asians. The use of ethnicity as a categorization variable is integral to the objectives of this research and is not used as a proxy for other socially constructed or relevant variables, such as socioeconomic status.

For clarity, we define the term "East Asians" in this study as encompassing two distinct groups: Southern Han and Northern Han. These designations were determined through robust genetic analysis methods, including Principal Component Analysis (PCA) and comparisons of the samples with 1000 genomic groups. The categorization into "East Asians" is based on the genetic characteristics exhibited by the study participants.

Population characteristics

The covariate-relevant population characteristics of the human research participants in this study are as follows. Please note that all calculations and data collection were performed with reference to December 31, 2021:

Age: Age of participants was calculated as of December 31, 2021, using the formula: Age = December 31, 2021 minus date of birth. This information provides an accurate snapshot of the age distribution within the study cohort.

Sex: The sex of participants was determined based on the SNP array genotyping result.

Diagnostic Information: Diagnostic data was determined based on the International Classification of Diseases (ICD) codes, including the 9th and 10th editions. Diagnostic information for all subjects was recorded and assessed up to December 31, 2021.

Genotypic: SNP array data were collected within the timeframe between the date of Institutional Review Board (IRB) approval and December 31, 2021. Genotyping was conducted using Axiom Genome-Wide 1.0 custom array plates provided by Affymetrix, Santa Clara, CA, USA.

Recruitment

Participants in this study were recruited exclusively from outpatient. It's important to acknowledge that this recruitment approach may introduce certain biases, primarily influenced by the flow of patients and the age distribution within various departments of the hospital.

As patients attending outpatient clinics are typically individuals seeking medical care for specific health concerns, the study cohort is inherently skewed toward individuals who require medical attention or ongoing treatment. This introduces a potential self-selection bias, as the study participants are not randomly drawn from the general population but rather represent a specific subgroup seeking medical services.

To mitigate these biases and enhance the generalizability of the results, careful attention was paid to study design and statistical analysis, with an emphasis on transparent reporting. Any limitations arising from the recruitment process have

been acknowledged, and the implications of these biases are considered when interpreting the results.

Ethics oversight	The study protocol for this research was subjected to thorough ethical oversight and received approval from the Institutional Review Board (IRB) with the following details: IRB number, CMUH107-REC3-058 (AR-1); date of approval, 07/20/2018

Note that full information on the approval of the study protocol must also be provided in the manuscript.

Field-specific reporting

Please select the one below that is the best fit for your research. If you are not sure, read the appropriate sections before making your selection.

☒ Life sciences ☐ Behavioural & social sciences ☐ Ecological, evolutionary & environmental sciences

For a reference copy of the document with all sections, see nature.com/documents/nr-reporting-summary-flat.pdf

Life sciences study design

All studies must disclose on these points even when the disclosure is negative.

Sample size	The determination of our sample size is based on several key considerations. The SNP array data from 300,000 individuals provides a substantial dataset, enabling us to screen suitable controls for individual phenotypes in our research.<br><br>The decision to screen phenotypes with more than 1,000 cases as research targets is not rigidly set but is guided by various factors. These factors include the specific research objectives, the complexity of the genetic traits under investigation, and the statistical power required to derive meaningful and robust conclusions.<br><br>Our sample size was chosen to strike a balance between the need for statistical power and the practicality of working with a large dataset. It allows us to address the research questions comprehensively, assess the impact of different variables, and draw reliable inferences based on the study's objectives. The size of the dataset, in this context, is considered sufficient for the purposes of this research.
Data exclusions	For the selection of cases and controls for each phenotype, we used the createPhenotypes function in PheWAS (https://github.com/PheWAS/PheWAS). The inclusion and exclusion criteria were established using the Clinical Classification Software grouping schema and the incidence of codes in several medical facilities' electronic health records, which are available at https://www.phewascatalog.org/phecodes.
Replication	While our study involved the statistical analysis of the application of PGS information from the PGS Catalog to CMUH's SNP array data, which differs from experimental research that typically requires replication, we ensured the reliability of our predictive power and generalization of models through the separation of our dataset into training and testing sets. At the beginning of the research, we used 10 cross validation to determine the stability of the model in the testing sets. When entering the statistical stage, we chose to fix the random variable to ensure that the model initialization was consistent for each experiment. This practice underscores our commitment to conducting rigorous and robust statistical research.
Randomization	To identify phenotypes for analysis, we obtained the ICD codes (International Classification of Diseases version 9th and 10th) recorded in medical records and patient demographics, such as age, sex, and other identifying information. We utilized the 'createPhenotypes' function within the PheWAS framework (available at https://github.com/PheWAS/PheWAS) to identify and group relevant phenotypes. These criteria were defined using the Clinical Classification Software grouping schema and were informed by the incidence of ICD codes within the electronic health records of multiple medical facilities. These records and criteria are accessible at https://www.phewascatalog.org/phecodes.
Blinding	Blinding in research refers to the process of withholding certain information from participants or researchers involved in a study to prevent biases from influencing the results. Blinding is typically relevant in experimental studies where participants are assigned to different groups (e.g., treatment and control groups) to assess the effects of interventions. The purpose of blinding is to minimize the potential for bias that could arise from participants or researchers knowing which group they are in.<br><br>In our study, which primarily involved the calculation and evaluation of polygenic scores (PGSs) across a wide range of phenotypes using genotyping array data, blinding of group assignment during data collection and analysis was not necessary or applicable. This is because our study was primarily retrospective and observational rather than experimental. We were not intervening or assigning participants to different groups for treatments or interventions.<br><br>Instead, our study involved the systematic calculation of PGSs across various phenotypes using existing genotyping array data. The data were collected in a manner that did not involve assigning participants to different groups or interventions. Furthermore, the analysis of PGSs and their predictive abilities for disease traits was conducted based on pre-existing data, without any manipulation or intervention during the study.

Reporting for specific materials, systems and methods

We require information from authors about some types of materials, experimental systems and methods used in many studies. Here, indicate whether each material, system or method listed is relevant to your study. If you are not sure if a list item applies to your research, read the appropriate section before selecting a response.

Materials & experimental systems

n/a	Involved in the study
☒	☐ Antibodies
☒	☐ Eukaryotic cell lines
☒	☐ Palaeontology and archaeology
☒	☐ Animals and other organisms
☒	☐ Clinical data
☒	☐ Dual use research of concern
☒	☐ Plants

Methods

n/a	Involved in the study
☒	☐ ChIP-seq
☒	☐ Flow cytometry
☒	☐ MRI-based neuroimaging
