# Supplementary figures and images for: Utility of polygenic scores across diverse diseases in a hospital cohort for predictive modeling
Source: Nat Commun. 2024 Apr 12;15:3168. doi: 10.1038/s41467-024-47472-5 (PMC11014845; doi:10.1038/s41467-024-47472-5)

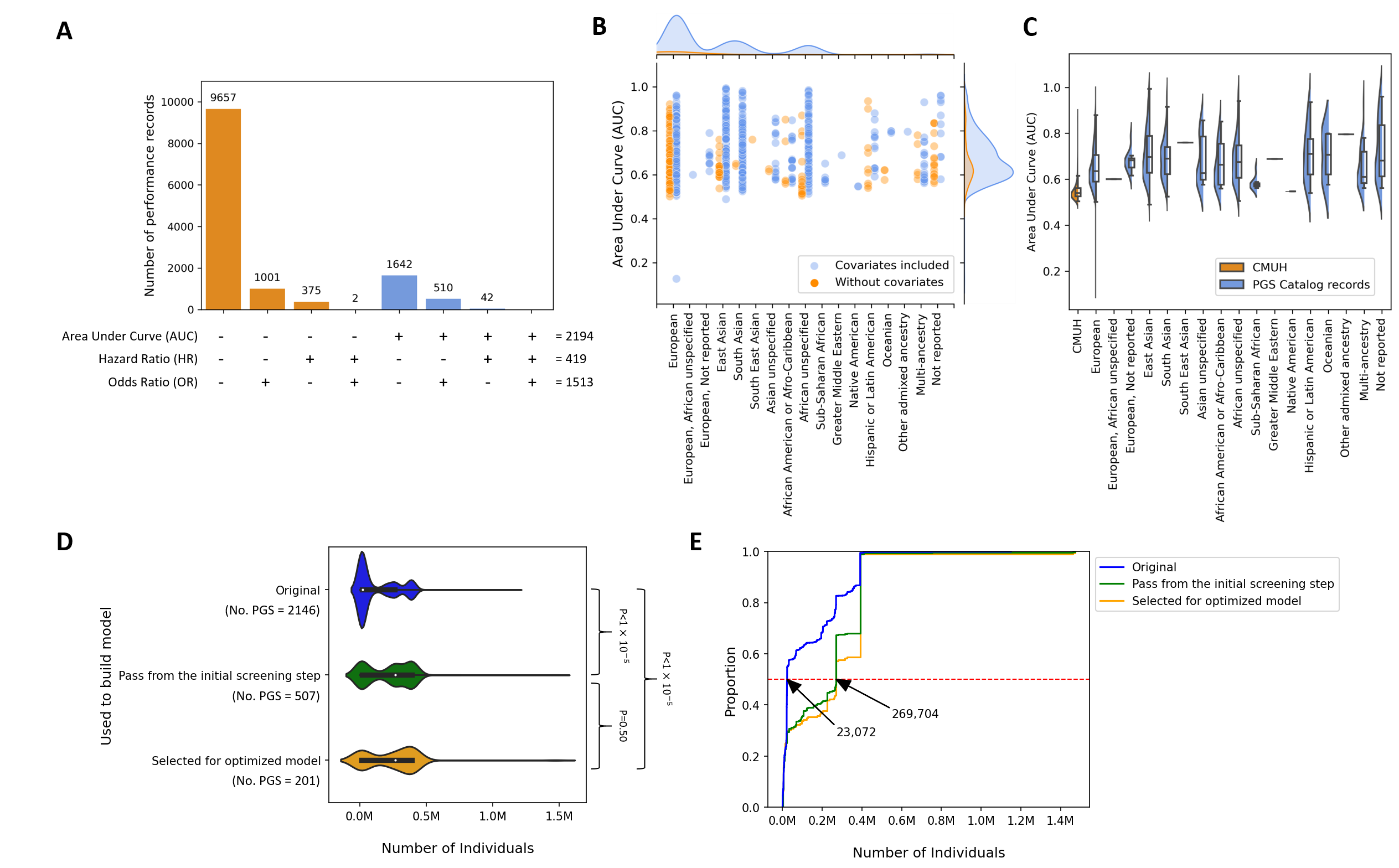

Supplement: Supplementary file 8 — Source Data [file 41467_2024_47472_MOESM8_ESM.zip › Source Data/Figure 1.tif]

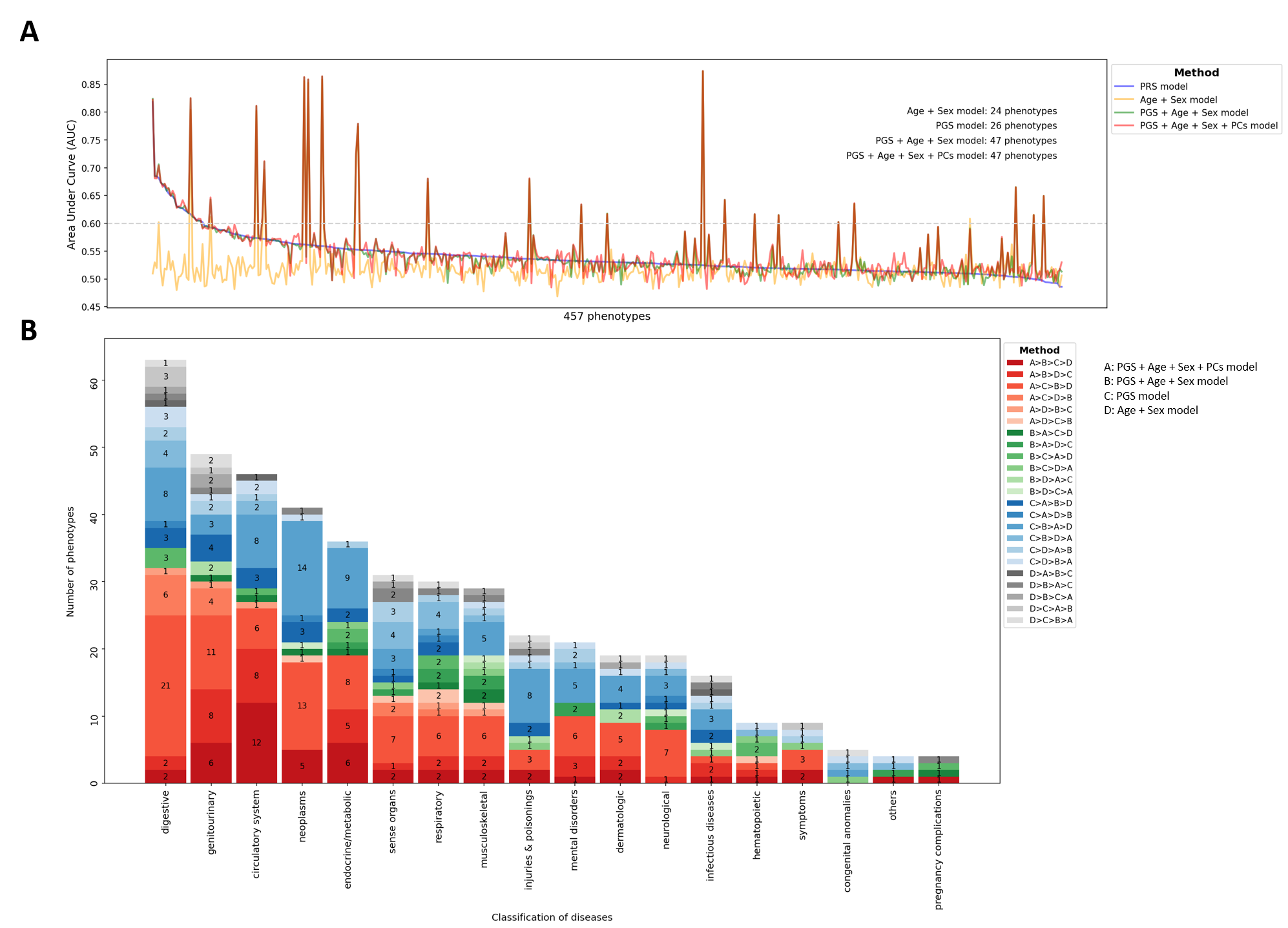

Supplement: Supplementary file 8 — Source Data [file 41467_2024_47472_MOESM8_ESM.zip › Source Data/Figure 2.tif]

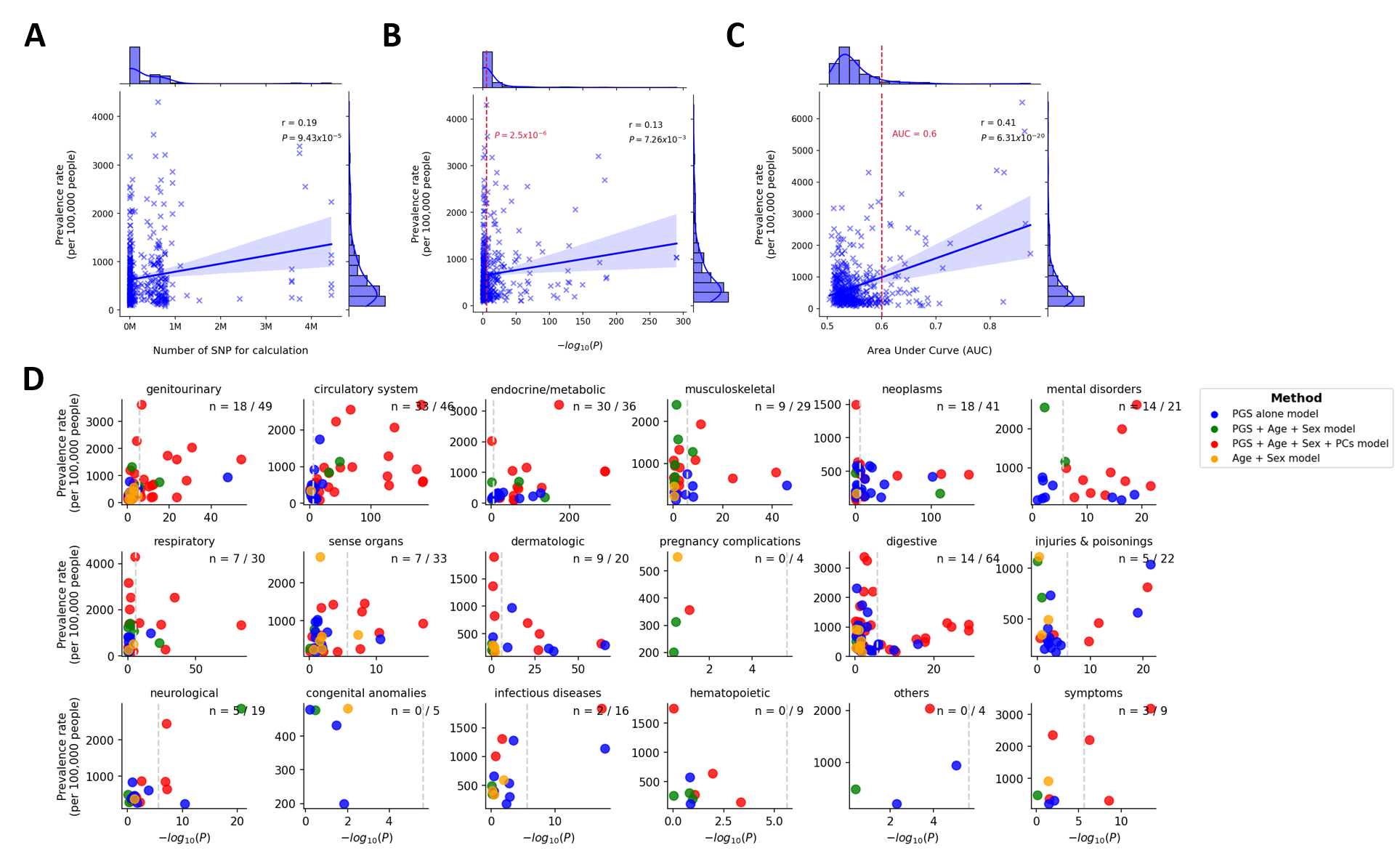

Supplement: Supplementary file 8 — Source Data [file 41467_2024_47472_MOESM8_ESM.zip › Source Data/Figure 3.tif]

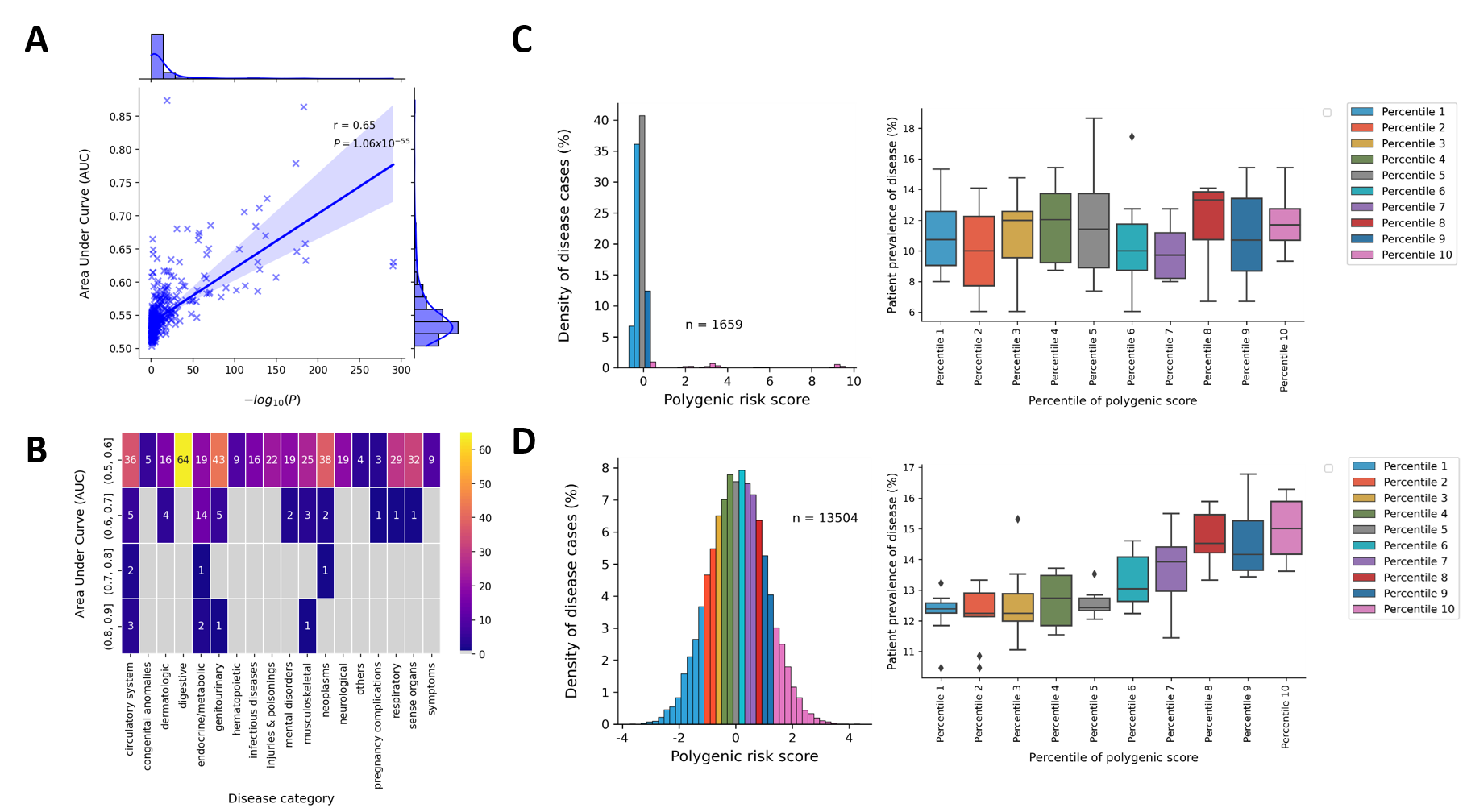

Supplement: Supplementary file 8 — Source Data [file 41467_2024_47472_MOESM8_ESM.zip › Source Data/Figure 4.tif]

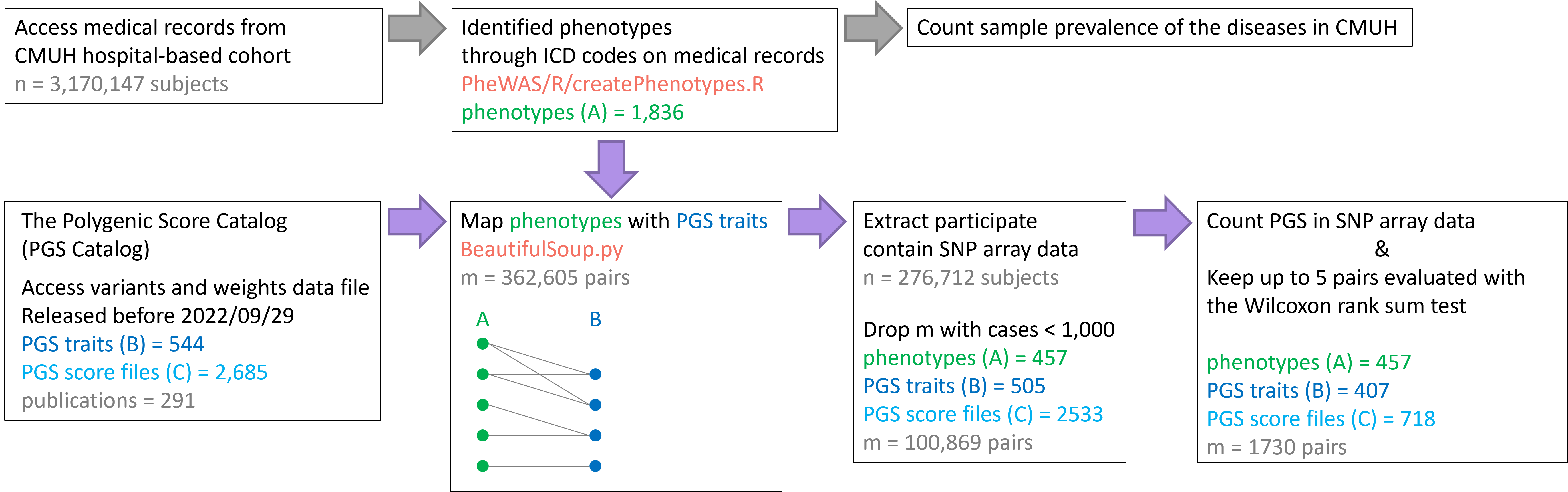

Supplement: Supplementary file 8 — Source Data [file 41467_2024_47472_MOESM8_ESM.zip › Source Data/Figure 5.tif]

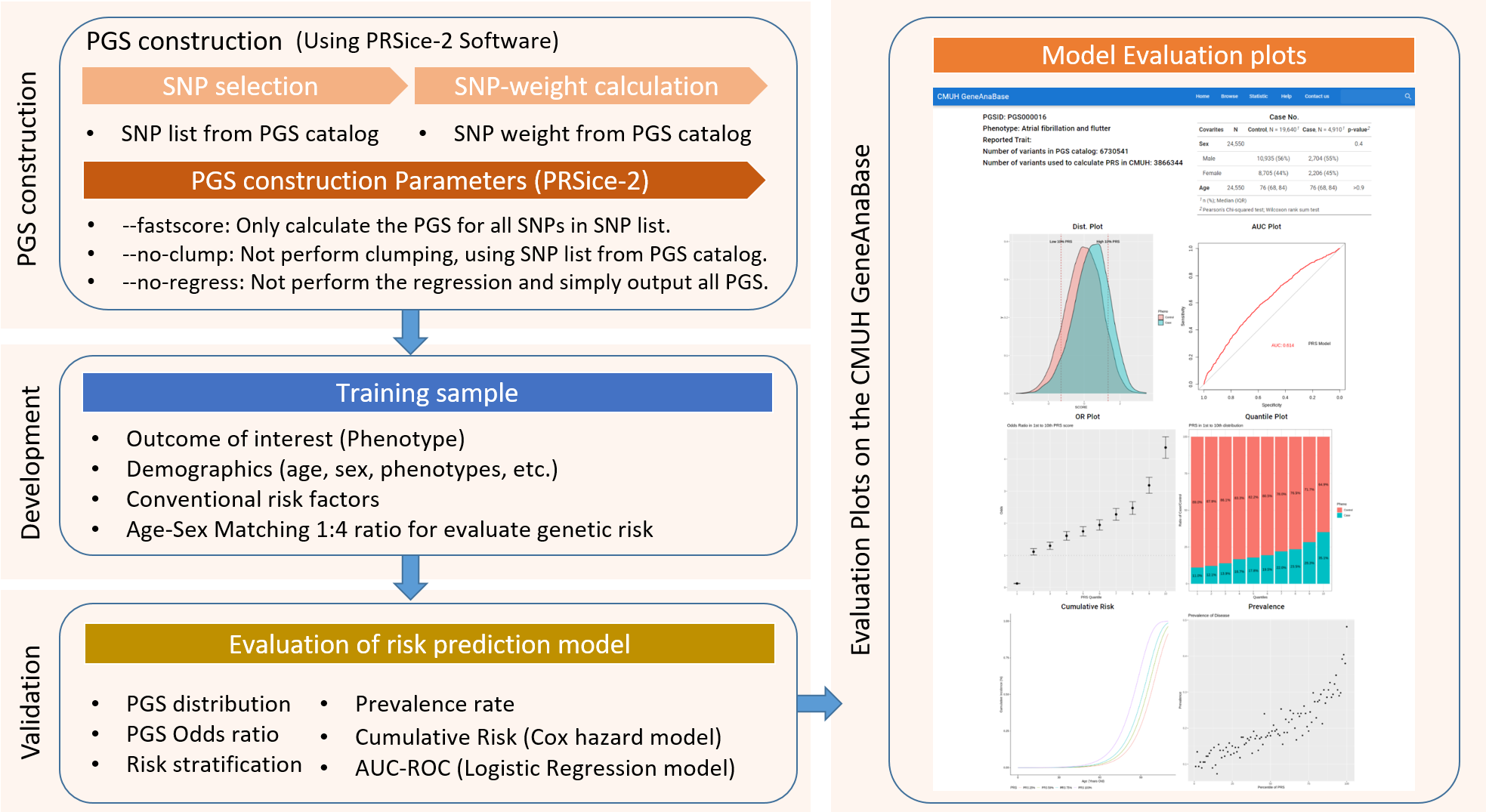

Supplement: Supplementary file 8 — Source Data [file 41467_2024_47472_MOESM8_ESM.zip › Source Data/Figure 6.tif]
